# Prevalence and biosocial determinants of hypertension in a rural population in Edo State, Southern Nigeria

**DOI:** 10.4102/phcfm.v13i1.2833

**Published:** 2021-07-01

**Authors:** Oghenekaro G. Egbi, Sulaiman D. Ahmed, Roli Madubuko

**Affiliations:** 1 Department of Internal Medicine, Niger Delta University Teaching Hospital, Okolobiri, Bayelsa State, Nigeria; 2 Department of Internal Medicine, Faculty of Clinical Sciences, Niger Delta University, Amassoma, Bayelsa State, Nigeria; 3 Department of Internal Medicine, Irrua Specialist Teaching Hospital, Irrua, Edo State, Nigeria; 4 Department of Internal Medicine, University of Benin, Benin City, Edo State, Nigeria

**Keywords:** blood pressure, body mass index, hypertension, obese, rural community

## Abstract

**Background:**

Hypertension is a public health threat of global concern with increasing prevalence in many countries, including Nigeria.

**Aim:**

The aim of the study was to determine the prevalence and determinants of hypertension in a rural agrarian community in Edo North, Nigeria.

**Setting:**

The study was carried out in Ayua, a community in Edo North, southern Nigeria.

**Methods:**

This cross-sectional descriptive study involved the use of a structured interviewer-administered questionnaire to obtain relevant data. Body mass index (BMI), blood pressure and glucose were recorded. Data were analysed using Statistical Package for Social Sciences (SPSS) version 20.0.

**Results:**

Two hundred and nineteen participants aged > 15 years completed the study with a mean age of 54.03 ± 16.61 years and females comprising 159 (72.6%) of the total. The prevalence of hypertension was 27.9% (in 61 participants). Twenty-one (9.8%) respondents gave a family history of hypertension. The mean BMI amongst respondents was 27.10 ± 6.61 kg/m^2^. Obesity and pre-obesity were found in 58 (26.5%) and 71(32.4%) respondents, respectively. The determinants of hypertension were age and BMI. Compared with those who were less than 40 years old, those aged 40–65 years and > 65 years had 1.9 and 4.2 times increased odds of developing hypertension, respectively. Similarly, compared with the non-obese, obese participants had 2.3 times increased odd of having hypertension.

**Conclusion:**

Hypertension was highly prevalent in this rural community. Health sensitisation and intervention programmes are recommended in rural communities for early detection and management of hypertension, especially amongst older and obese adults.

## Introduction

Globally, hypertension is a common public health threat, often resulting in devastating systemic complications such as cerebrovascular accident, kidney and heart failure, and blindness. Cardiovascular disease accounts for approximately 17 million deaths a year, about one-third of total deaths worldwide^1^ with complications of hypertension responsible for 9.4 million deaths yearly.^2^ This is without prejudice to the asymptomatic nature of its uncomplicated form which has earned it a unique nomenclature ‘the silent killer’.^3^

Hypertension affects approximately 1.13 billion people worldwide^4^ and only less than 20% of affected persons have the problem under control.^5^ Contrary to the popular belief that hypertension is more prevalent in high-income countries, it has been shown that three-quarters of the world’s hypertensive population now reside in low- and middle-income countries.^6^ In Nigeria, a systematic review reported prevalence rates ranging from 9.3% in Ibadan^7^ to 50.5% in Ogbomosho^8^ with higher rates in urban communities. Ulasi et al reported a prevalence of 25.1% and 35.4% in a rural and semi-urban community, respectively, in South-East Nigeria.^9^ However, it appears the existing gaps between rates in urban and rural communities are gradually closing up. Recent reports across Nigeria suggest an increasing prevalence amongst rural populations.^10^ This may not be unrelated to nutritional transition, changes in lifestyle, and socio-cultural patterns in rural settings.^11^ Other non-communicable diseases which often cluster with hypertension such as obesity and diabetes have also been on the rise in such communities.^11^ One could however hypothesise that non-sedentary occupations such as farming, common in rural settings, would confer some protection against the risk of developing hypertension. The aim of the study was to determine the prevalence and some determinants of hypertension amongst residents of Ayua, a rural agrarian community in Edo North, Nigeria. It is expected that community screening for hypertension could help in timely detection of silent hypertension and associated factors. This will be important for strategic health planning with a goal of early intervention to mitigate the risk of complications.

## Methods and design

### Study design

The study employed a descriptive cross-sectional design and a quantitative method of data collection. The entire study including the conceptual design, training of the research team, the screening exercise and data storage took place from February 2015 to May 2015.

### Study setting

The study was carried out in Ayua, a rural community located in Etsako West local government area in Edo North Senatorial District of Edo State. Edo is one of the six states in the South–South geopolitical zone of Nigeria. Ayua has a total population of 3058 persons with 1570 females constituting about 51.3% of the population.^12^ The indigenous language spoken by the community is ‘Afemai’ also known as ‘Yekhee’ whilst the major occupation of the inhabitants is subsistence farming. The usual crops grown in the community include rice, yam, cassava, melon, beans and palm produce. They are also involved in livestock farming such as poultry, goat and sheep rearing. Their staple diet includes rice, cassava meal and yam.

### Study population and sampling strategy

The study population consisted of consenting adults who met the criteria for inclusion. The inclusion criteria included all adults > 15 years who showed willingness to participate in the study. Pregnant women, very ill persons, individuals unable to stand, and those who were uncooperative were excluded from the study. Also excluded were individuals with a history of steroid use, secondary causes of hypertension such as renal disease, clinical evidence of fluid retention or other overt complications of hypertension such as stroke or heart failure.

The sample size for the study was calculated using the Fisher’s formula for finite population as follows:

$$n_{0}=(z^{2}p[1-p])/d^{2}\text{ and }n=n_{0}N/n_{0}+(N-1)$$ [Eqn 1]

Here,

*n*_0_ = sample size for infinite population; *n* = sample size for finite population; *p* = prevalence of hypertension; d = precision set at 0.05.

The prevalence of hypertension in Udo, a community in Ovia local government of Edo State, reported as 20.2% was used in calculation of the sample size.^13^

Substituting for ‘*p*’ in the formula gives:

$$n_{0}=1.96\times 1.96\times 0.125\times (1-0.125)/0.05\times 0.05-151.9=248$$ [Eqn 2]

$$n=n_{0}N/n_{0}+(N-1)=151.9\times 1570/151.9+(1570-1)=229$$ [Eqn 3]

Adding 10% of ‘*n*’ for non-response or incomplete data results in a sample size of 252. Therefore, a total of 250 persons were enrolled for the study.

A convenient sampling of individuals who presented for the health survey at the designated venue, on scheduled date and time, was done until the desired sample size was achieved.

### Study procedure and data collection

The study involved an initial health sensitisation and advocacy in the community. The traditional rulers, leaders of the various religious organisations and trade unions were consulted and given prior notice to mobilise their subjects for the health screening exercise which was to take place on a particular weekend in April 2021 in the village town hall. Data collection was done over a period of about 8 h.

The research team was made up of two consultant physicians (one of whom was the principal researcher and head of the team), five medical officers, eight nurses, three pharmacists, two laboratory scientists and two other research assistants. They had a 3-day training by the principal researcher on the questionnaire and other relevant aspects of the study just prior to the commencement of the study. An interviewer-administered questionnaire was developed and divided into two sections. Section A contained information on demographics (including age and gender) and information on medical history (personal history of hypertension and diabetes and family history of hypertension and diabetes) whilst anthropometric data and data on blood pressure and glucose were contained in Section B. The screening exercise was preceded by a health talk on hypertension given by one of the medical doctors in pidgin and translated to the local dialect by a native of the community. Two doctors were responsible for obtaining the socio-demographic data and medical history from the participants. The nurses were responsible for blood pressure check and anthropometric measurements. The ‘other research assistants’ were responsible for coordination of the overall activities and orderliness of the exercise. Blood sampling and analysis for plasma glucose were performed by the laboratory scientists. Patients found to be hypertensive, diabetic or obese were also referred to the other doctors who counselled and prescribed medications where needed. The medications were dispensed by the pharmacists. Such patients were subsequently referred for follow up in a nearby health facility.

Blood pressure was measured using an OMRON digital sphygmomanometer (OMRON, Kyoto, Japan). An average of two readings was taken at 5 min interval. The individual was required to sit quietly on a chair and should not have taken caffeine, nicotine or alcohol and must also not have exercised at least 30 min prior to measurement. The systolic blood pressure (SBP) was taken as the point at which the first of the Korotkoff sounds was heard whilst the complete disappearance of Korotkoff sounds was used to define diastolic blood pressure (DBP).

Participants’ weights were measured on a manual bathroom scale to one decimal figure in kilograms after removing heavy clothing and shoes and putting aside phones and other items. Similarly, a marked ‘height wall’ was used to measure height to the nearest 0.01 m with the participant erect, backs against the wall and feet placed side by side without shoes. The body mass index (BMI) was computed by dividing the weight by the square of the height and expressed in kg/m^2^.

Casual blood glucose (CBG) measurement was done with an Accuchek glucometer using capillary blood from finger prick according to standard procedures.

### Operational definitions

Hypertension was defined as a measured blood pressure ≥ 140 millimetre of mercury (mmHg) systolic and/or ≥ 90 mmHg diastolic or self-reported use of drug treatment for hypertension irrespective of measured blood pressure.^14^

Hypertensives were participants with SBP > 140 mmHg and/or DBP > 90 mmHg and/or a personal history of hypertension whilst non-hypertensives were participants with SBP < 140 mmHg, DBP < 90 mmHg and without a personal history of hypertension.

A personal history of hypertension was defined as a self-report of diagnosis of hypertension whilst a family history of hypertension was defined as a diagnosis of hypertension in first- or second-degree relatives.

Diabetes mellitus (DM) was defined in this study as CBG of ≥ 11.1 mmol/L with a history of suggestive symptoms.^15^

A personal history of DM was defined as a self-report of diagnosis of diabetes amongst participants. A family history of DM was defined as a prior diagnosis of DM in first- or second-degree relatives.

Obesity was defined as BMI > 30 kg/m^2^ whilst pre-obesity was defined as BMI > 25 kg/m^2^ but less than 30 kg/m^2^. Normal BMI was defined as values between 18.5 kg/m^2^ and 24.9 kg/m^2^ whilst lesser values were considered as ‘underweight’.^16^

### Data analysis

The software used for the data analysis was the Statistical Package for Social Sciences (SPSS) International Business Machines (IBM) statistics for Windows version 20.0. Descriptive analysis was done to describe the demographic and clinical characteristics of the participants such as age and the presence of a personal or family history of hypertension and diabetes. Prevalence of hypertension was estimated by determining the proportion of individuals who met criteria for definition of hypertension compared with the total population. The chi-square test was used to compare the proportions of categorical variables of hypertensives and non-hypertensives. Where the variables were continuous, a student *t*-test was employed. To identify the determinants of hypertension, multivariate logistic regression analysis was performed using hypertension as the dependent variable and age, gender, BMI, personal history of diabetes, family history of diabetes and hypertension and the presence of diabetes as the independent variables with adjusted odds ratio and confidence intervals computed. For all inferential analysis, a *p*-value of < 0.05 was considered significant. Results were presented in tabular and graphical forms.

## Results

Two hundred and fifty persons were selected to participate in the study but only 219 (87.6%) had complete data useful for analysis. The socio-demographic data of respondents is shown in Table 1. The number of females (*n* = 159, 72.6%) was remarkably greater than the males (*n* = 60, 27.4%). The mean age of the respondents was 54.03 ± 16.61 years with no significant difference between males and females (*P* > 0.05). Seventy one (33.0%) individuals had normal BMI whilst 48 (26.5%) were obese. A history of hypertension and diabetes was given in 23 (10.5%) and 18 (8.2%) respondents, respectively. Whilst a family history of hypertension was found in 21 (9.8%) persons, 18 (8.2%) had a family history of DM.

**TABLE 1:** Clinico-demographic variables of participants.

Variable	Mean ± s.d.	Range	Frequency	
			*n*	%
**Age (years)**	54.03 ± 16.61	15–90	-	-
**Age group**	-	-	-	-
< 40	-	-	57	26.0
40–65	-	-	110	50.3
> 65	-	-	52	23.7
**Gender**	-	-	-	-
Male	-	-	60	27.4
Female	-	-	159	72.6
**BMI (kg/m^2^)**	27.10 ± 6.61	11.92–55.38	-	-
**BMI category**	-	-	-	-
Underweight	-	-	11	5.0
Normal	-	-	79	36.1
Pre-obese	-	-	71	32.4
Obese	-	-	58	26.5
**Personal history of high blood pressure**	-	-	-	-
Present	-	-	196	89.5
Absent	-	-	23	10.5
**Family history of high blood pressure**		-	-	-
Present	-	-	21	9.6
Absent	-	-	198	90.4
**Personal history of high blood glucose**	-	-	-	-
Present	-	-	18	8.2
Absent	-	-	201	91.8
**Family history of high blood glucose**		-	-	-
Present			18	8.2
Absent		-	201	91.8

s.d., standard deviation; BMI, body mass index.

The hypertensives were older with a mean age of 60.71 ± 15.13 years compared with the non-hypertensives who had a mean age of 52.30 ± 16.57 (*p* = 0.002). They also had a higher mean BMI (28.98 ± 8.05) compared with the non-hypertensives (26.61 ± 6.11), *p* = 0.03 (Table 2). However, there was no statistically significant difference between the presence of a family history of hypertension or family history of diabetes and presence of hypertension (*p* = 0.13 and 0.09, respectively). Whilst four (6.6%) hypertensives had an associated DM, nine (5.7%) non-hypertensives had DM. There was no significant difference in the association of hypertension with DM (*p* = 0.758). Also, although the mean CBG was higher for hypertensives (7.05 ± 5.63 mmol/L) as compared to non-hypertensives (6.71 ± 4.48 mmol/L), there was no statistical significance between them (*p* = 0.676) (see Table 2).

**TABLE 2:** Comparison of characteristics of hypertensive and non-hypertensive participants.

Variable	HTN		*t*	*p*	HTN				*X*^2^	*p*
	Yes	No			Yes		No			
	
	Mean ± s.d.	Mean ± s.d.			*n*	%	*n*	%		
**Age(years)**	60.71 ± 15.13	52.30 ± 16.57	−3.087	0.002	-	-	-	-	-	-
**Gender**										
Male	-	-	-	-	14	23.3	46	76.7	0.393	-
Female	-	-	-	-	31	19.5	128	80.5	-	0.575
**Body mass index (kg/m^2^)**	28.98 ± 8.05	26.61 ± 6.11	−2.159	0.03	-	-	-	-	-	-
**Family Hx of high blood pressure**										
Present	-	-	-	-	1	4.8	20	95.2	3.545	-
Absent	-	-	-	-	44	22.2	154	77.8	-	0.085
**Personal Hx of high blood glucose**										
Present	-	-	-	-	2	11.1	16	88.9	1.0	-
Absent	-	-	-	-	43	21.4	158	78.6	-	0.379
**Family Hx of high blood glucose**										
Present	-	-	-	-	1	5.6	17	94.4	2.700	-
Absent	-	-	-	-	157	78.1	44	21.9	-	0.131
**Casual blood glucose**	7.05 ± 5.63	6.71 ± 4.48	−0.419	0.676	-		-		-	-
**Diabetes**										
Present	-	-	-	-	4	6.6	9	5.7	0.058	-
Absent	-	-	-	-	57	93.4	149	94.3	-	0.758

HTN, hypertension; Hx, history; *n*, frequency; s.d., standard deviation; *X*^2^, chi-square.

The prevalence of hypertension was 27.9% (present in 61 persons). Systolic blood pressure of participants ranged from 70 mmHg to 220 mmHg with a median of 130 mmHg whilst DBP ranged from 40 mmHg to 110 mmHg with a median of 80 mmHg. The mean SBPs for males and females were 133.76 ± 17.67 mmHg and 129.27 ± 21.53 mmHg, respectively, whilst the mean DBP for male and female participants were 78.96 ± 12.55 mmHg and 78.77 ± 12.35 mmHg, respectively. Although the mean blood pressures were higher in males, statistically significant difference was not met (*p* = 0.151 and 0.919 for SBP and DBP, respectively; see Table 3).

**TABLE 3:** Comparison of blood pressure between male and female participants.

Blood pressure (mmHg)	Total	Male	Female	*p*
**Systolic blood pressure**				
Range	70–220	100–200	70–220	-
Mean ± s.d.	130.50 ± 20.08	133.76 ± 17.67	129.27 ± 21.53	-
Median	130.00	130.00	130.00	0.151
SEM	1.393	2.282	1.708	-
**Diastolic blood pressure**				
Range	40–110	40–110	40–100	-
Mean ± s.d.	78.83 ± 12.38	78.96 ± 12.55	78.77 ± 12.35	-
Median	80.00	80.00	80.00	0.919
SEM	0.836	1.621	0.979	-

SEM, standard error of the mean; s.d., standard deviation.

**FIGURE 1:**
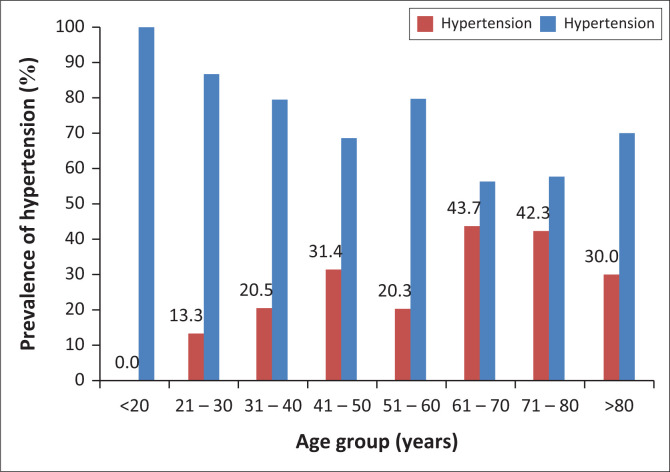
Prevalence of hypertension related to age groups of participants.

**FIGURE 2:**
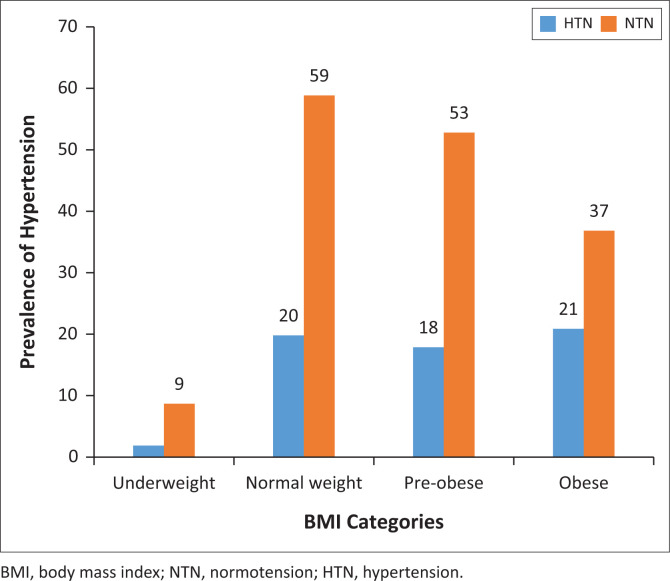
Prevalence of hypertension related to body mass index of the participants.

The result of multivariate analysis to identify determinants of hypertension is shown in Table 4. Hypertension was independently predicted by age and BMI in this study. Compared with those who were less than 40 years old, individuals aged 40–65 years and those > 65 years old had 2.3 and 4.2 times increased odds of developing hypertension. Similarly, obese participants had a 2.3 times increased odd of developing hypertension (Table 4). The association of hypertension with age and BMI is further represented in Figures 1 and 2, respectively. The highest prevalence of hypertension was found in those within the range of 61–70 years (43.7%). There was no (0.0%) hypertensive individual amongst those less than 20 years old. There was a gradual increase in prevalence rates until age 60. (Figure 1) The prevalence of hypertension in underweight, normal, pre-obese and obese individuals were 18.2% (2/11), 25.3%, (20/79), 25.4% (18/71) and 36.2% (21/58), respectively (Figure 2).

**TABLE 4:** Factors associated with hypertension in multivariate logistic regression.

Variable	OR	95% CI	*p*
**Age group**			
< 40	1.00	-	0.024
40–65	1.943	0.908-6.105	0.078
> 65	4.240	1.493-11.705	0.006*
**Gender**			
Female	1.00	-	-
Male	1.558	0.738-3.287	0.244
**BMI category**			
Not obese	1.00	-	-
Obese	2.266	1.081-4.752	0.030*
**Family history of HTN**			
Absent	1.00	-	-
Present	0.657	0.055-7.837	0.740
**Personal history of DM**			
Absent	1.00	-	-
Present	13.150	0.833-207.637	0.067
**Family history of DM**			
Absent	1.00	-	-
Present	3.159	0.203-49.091	0.411
**Diabetes**			
Absent	1.00	-	-
Present	1.005	0.258-3.911	0.994

HTN, hypertension; BMI, body mass index; OR, odds ratio; CI, confidence interval; DM, diabetes mellitus.

* , statistically significant.

## Discussion

This study reports a high prevalence of hypertension (27.9%) in a rural, agrarian community in Edo State, Nigeria. This is consistent with reports from most communities in Nigeria with rates ranging from about 20.8% to 46.4% over the last decade.^17,18^ In the latter study,^18^ Onwubere et al. may have found a much higher rate because those less than 40 years old were excluded in their study. More lately, Akpan et al also reported a relatively high prevalence of 44.3% in rural communities compared with 28.6% in urban communities in Akwa Ibom, Nigeria.^13^ This is in contrast to an earlier comparative study done in year 2013 between urban and rural communities in Abuja, the Nigerian Federal Capital Territory, which gave a disproportionally lower rate in the rural community (12.9%) as against the urban area (32.7%) studied.^19^ However, in a similar rural setting predominated by farmers, just like ours, Ugwuja et al. reported a slightly lower prevalence of 23.2% in Enugu State, Nigeria, in 2015.^20^ However, their study did not incorporate those with a history of hypertension, but with normal measured blood pressure, into the diagnosis of hypertension and this may have contributed to the difference in the rates.

The age-specific prevalence of hypertension was highest in the age group 61–70 years, having increased gradually from 0.0% in those below 20 years till age 50. There was up to four times increased odds for hypertension amongst those above 65 years of age compared with adults who were below 20 years old. Similarly, the obese had double the risk of development of high blood pressure in comparison with those with normal BMI. These findings are in agreement with reports which have alluded to strong positive association of blood pressure with age and BMI. In fact, several studies considered the duo of age and BMI as the most significant predictor of hypertension in Nigeria^20,21,22^ and elsewhere.^23,24^ Firstly, the association of obesity with hypertension is important; as obesity is modifiable, it could have a strategic place in management of hypertension. Secondly, the prevalence of obesity is on the increase, and is presently assuming epidemic dimensions in several countries including Nigeria.^25^ This has important implications for the burden of hypertension. Apart from BMI, regional fat distribution has also been reported as a major determinant of blood pressure.^26,27^ A meta-analysis conducted in West Africa also included gender alongside obesity and age as predictors of hypertension.^28^ However, we did not find any association between hypertension and gender. The female participants largely outnumbered the males in our study even though the population was only slightly tilted in their favour. This is not surprising as it is believed that Nigerian women display a better health-seeking behaviour as compared to their male counterparts.^29^ There was however no difference in the demographic characteristics or blood pressure indices between males and females. Our results agree with the findings of another large meta-analysis involving both low- and medium-income countries that failed to demonstrate a significant association of hypertension with gender.^30^ The determination of the exact influences of gender on blood pressure is still inconclusive and warrants further studies. In another report from Delta State, Nigeria, increasing age, BMI and salt intake were independently associated with hypertension.^31^ However, we did not assess for dietary factors in our study.

A family history of diabetes and plasma glucose were reported as determinants of hypertension in the Ibadan study.^26^ We however did not find an association between any of these variables and hypertension. In our study, we recorded CBG values whilst in theirs, a 2 h plasma glucose level was assessed after an oral glucose loading. Also, the cut-off for hypertension (160/95 mmHg) used in that study was different from ours.

Although the proportion of hypertensives with a family history of hypertension was remarkably more compared with hypertensives without a family history, statistical significance was not reached. This is similar to the report of Lebbie et al where the association between family history of hypertension and blood pressure was only close to conventional level of significance (*p* < 0.1 but not > 0.05).^23^ A number of studies have however reported that a family history of hypertension could predict hypertension in relatives.^32,33^ It is important to note that such self-report of history of illness or diagnosis without any objective confirmation may be prone to report or recall bias. Other limitations of this study include failure to explore certain risk factors which have long been documented to be associated with hypertension such as physical inactivity, tobacco and alcohol use.^34^ The convenience sampling technique used in this study, being a non-probability sampling method, may pose some challenges on the extent to which the sample is representative of the population.

Despite these limitations, the findings of a high prevalence of hypertension in this rural community and its predictability by age and BMI remain valid. It is likely that the rate of hypertension is on the increase in rural populations. Routine screening for hypertension in such settings with subsequent intervention is highly recommended especially amongst older people with associated obesity.
